# Letter from Prof. J. P. White

**Published:** 1851-07

**Authors:** James P. White

**Affiliations:** Paris


### ART. III.—*Letter from Prof. J. P. White.*

*Paris, April* 10, 1851.

MY DEAR DOCTOR,—Since my last letter to you I have attended, among others, the lectures of M. Paul Dubois, at *La clinique*, which are delivered upon the cases possessing most interest, after the visits, on Tuesday, Thursday and Saturday mornings. M. Dubois is the son of the celebrated Antoine Dubois, and the "clinical professor of accouchments in the faculty of medicine, of Paris." To him is conceded the front rank as an obstetrician in this country, and he may be considered without doubt, the first living operator and practitioner in his department. He is rather above medium heighth, slightly stooping, quite bald, with an expressive benevolent countenance, and apparently about sixty-five years old. He has suffered severely with the epidemic recently prevailing here, (the grippe,) but his health is ordinarily good, and he is vigorous and active in his professional pursuits. Notwithstanding his immense private practice, and although entirely independent in his pecuniary condition, he continues his connection with the Hospitals Maternity and La Clinique, thus adding several hours of severe labor daily to his other arduous duties. In addition to all which he makes frequent contributions in the way of essays or papers upon subjects connected with his chair, at the weekly meetings of the Academy of Medicine; and still finds time to respond to the demands which his large domestic circle and general society make upon him. As the services at the hospitals are rendered for a mere nominal compensation, and can scarcely add to the reputation which he has already acquired, it may be safely concluded that in continuing these voluntary labors he is prompted mainly by a love of science. By keeping alive his zeal for scientific pursuits in the midst of his other avocations, as he advances in years, what a praiseworthy instance does he afford of industrious perseverance in these investigations, and from the most laudable motives!

With the mature and judicious practical good sense which M. Dubois possesses in an eminent degree, superadded to his extended acquirements, it is not a matter of surprise that the publication of a work by him which shall give to the world the full benefit of his observations, should be regarded with especial favor. Nor is the desire to see his views upon all subjects connected with obstetric science fully set forth by his own hand, diminished by the foretaste which is afforded in the works of his pupils M. M. Chailly, and Cazeau. It is here well understood that whatever merit these gentlemen may of themselves possess, for a very large proportion of the valuable matter which their writings contain they are indebted to his clinical instructions whilst they successively occupied the position of "*chef de clinique.*" A *livraison* containing 270 pages, the first of a promised series of eight, has appeared, and fully answers the high expectations which had been excited by the reputation of the author. This portion of the work is occupied with a full description of the organs of the female concerned in the functions of generation, and, so far as he has progressed, it leaves little to be wished. Even upon these trite subjects there are to be found new ideas. The various parts are described with great fullness and perspicuity, and the text is embellished with numerous well designed and well executed engravings. In running it over I observe that the anatomy and functions of the female breast are much more minutely given than in any of the standard works with which I am familiar, and the important part which that organ performs in the process of reproduction plainly and fully set forth. In his introduction to the first volume, M. Dubois takes occasion to speak of his estimate of the value of clinical instructions in this department of medical science, and, believing you will appreciate them, I copy some of his remarks upon this subject as literally as I am able to translate them. "Midwifery is one of the most important departments of general medicine. Its objects are necessarily complex, inasmuch as two beings are interested at the same time, having therefore a double mission to perform. This end can only be fully attained by the aid of anatomy, physiology, pathology, and therapeutics. * * * Unfortunately, the scope of teaching in this country has been too restricted. Far from taking an enlarged and philosophical view of the whole subject, it is confined to the narrow circle of the phenomena of parturition. Mechanical theories are adopted, disregarding the laws of physiology, which are quite as important. For a long time, if not the only, at least the essential and predominant objects of obstetrical studies, have consisted in a small number of operations or manœuvres of doubtful utility, being demonstrated upon the bony pelvis or upon manikins. To such an extent has the mind of the profession been preoccupied in this respect, that traces of it are still to be met with in our books and schools. One ought not to be surprised that even at this day, in the study of a great number of physiological and pathological phenomena which pertain to obstetric medicine, it should be found difficult to distinguish the real difference originating in actions purely organic; and perhaps more difficult yet to make even some eminent men comprehend the rational extent of a scientific domain so long obscured and contracted by those whose mission it was to enlighten and enlarge, It is probable that clinical teaching of the art of accouchments, too long incomplete among us, will contribute, more than any thing else, to the accomplishment of this work, not alone by any personal efforts of the individual, but by force of circumstances. For it is certain that it is impossible to collect in an establishment devoted to humanity and science pregnant females, women in labor, women recently delivered, and their infants, without, when submitted to an intelligent mind, suggesting questions as numerous, as various, as grave, as delicate, at least as important to the interests of those concerned, and, in fine, of an order as elevated as any of those which are raised and discussed in all other clinical teaching." It gives me pleasure to be able to add, that M. Dubois assures me, notwithstanding his numerous other engagements he hopes soon to have another volume ready for the press.

Having occasion to auscult a pregnant female in one of the wards a few days since, he diagnosed twins, (which I may add has since been verified) and then in his lecture took occasion to call the attention of the students to the value of the stethoscope in obstetric practice. He also gave his views upon the manner in which the *second sound* is produced. I refer to the *souffle,* which is termed by Kennedy and others the placental sound, or souffle. You are doubtless aware that the source of this sound has been the subject of great speculation. Upon its origin and mode of formation must depend the value of the practical deductions derived from it; and it is therefore important that this point should be established. Much as this result is to be desired, it must be admitted, that, although the doctrine of its placental origin seems overthrown, there is far from being, at this time, uniformity of opinion as to its mechanism and seat.

M. Kergaredec, who was the first to call the attention of the profession to this sound, seems to have felt much uncertainty in relation to its precise location. He evidently inclined to the belief that it would be found in that region of the uterus to which the placenta is attached. Simply remarking that it was produced by certain arteries which are dilated during pregnancy, and which permeate the uterine parieties, he did not undertake to explain the mechanism of this phenomena.

Hans located this souffle in the abdominal aorta, or iliac arteries, and believed it might often be heard throughout the entire uterine globe. M. Bouillaud advocated this opinion, compared it to the sound produced by compressing a large artery, said it might be produced by tumors developed in the abdomen of the non-pregnant female, and that its characters might be modified or made completely to disappear by changing the position of the subject under examination. Cassuron attributed it to the passage of the blood of the fœtus through the hole of Botot. Kennedy made it depend upon the utero-placental circulation; Monod, upon the placental circulation alone; Hall, upon the passage of the arterial blood into the maternal portion of the placenta. The latter rely for the support of their location of its seat chiefly upon the supposed coincidence of the souffle with the point of attachment of the placenta.

It is to M. P. Dubois that we are indebted for some interesting researches upon this obscure point. He first established the position that it must exist in the uterine parietes; and, in his own opinion, he also establishes the farther point, that the sound is not limited to the point of placental insertion. The placenta is ordinarily inserted near the fundus of the uterus, whilst the souffle exists in general towards the inferior part of the organ, and may be encountered throughout all its regions. "If," he says, "we admit, with the majority of observers, that this phenomenon depends upon placental connection, it ought to diminish in proportion as the vascular connection between the uterus and placenta are interrupted, and ought to disappear completely when this connection is destroyed, whereas, he adds, it may be heard, not only to the termination of labor, but, in some cases, after the expulsion of the placenta, when it would be impossible that it could be produced by that organ. It ought also, if dependent upon the placenta, or its vessels, to be heard only at the point of its attachment, whereas it may often be heard upon both sides at the same time, and at the inferior origin of the uterine globe, whilst that organ is ordinarily attached at the fundus. In some cases also it may be heard with more or less distinctness throughout the uterine tumor in the same examination." After much investigation he concludes that this sound resides in the uterine parieties themselves, and as the walls of the region to which the placenta is attached has a vascularity peculiar to itself, it may be the point from which it radiates, and some reasons may be urged for admitting that this is ordinarily true. As to the mechanism which developes this sound, he compares it both in the kind of sounds, as well as manner of production, to aneurismal varices, where the blood in passing from one class of vessels into another, gives rise to a sound exceedingly analogous to that under consideration. It was this supposed similarity of the two sounds which induced him to search for a similarity of cause in the vascular apparatus of the uterus as developed during pregnancy. He feels warranted, from injections made with different liquids, and with air, in declaring, that direct and numerous communications exist between the arteries and veins of the developed uterus; in short, that its parietes seem to be formed of a tissue or plexus of "*anevrismes variqueux naturels*," and that the column of blood carried by the arteries, and ramifying in their several branches, goes to be mixed in passing directly into the veins, with the less rapidly circulating, less forced columns, contained in these vessels. That there takes place then a direct mixture of the red arterial current with the dark venous blood which does not circulate with the same rapidity as that flowing in the arterial branches. In fine, he would call this sound, (and with great propriety if this hypothesis be true,) the "*uterine souffle*," believing that it has not its origin in the vessels of the placenta, but in the vascular apparatus of the uterus, although it may have its maximum of intensity at the point of attachment of the former organ, because there the vascular tissue of the latter is most developed, though not limited to that region, being often observed in points of the uterus which have no connection with the placenta. That it is determined by the same causes which produce the "*bruit de souffle*," in aneurismal varices, that is to say, by the direct passage of the arterial blood into the venous system, and by the mixing of the liquid columns which, at the moment of contact, have neither the same direction or rapidity of motion.

This theory, with slight modifications, is adopted by the principal teachers of obstetrics in this metropolis. M. Depaul, one of the pupils of M. Dubois, who has published a work upon the subject of "obstetric auscultation," coincides entirely with the opinions just advanced in relation to its seat, and thinks it liable to change its position in consequence of any change of pressure by the parts of the fœtus: that it exists in the vascular system of the uterine parieties when uninterrupted by pressure, but when the prominent points of the fœtal ovoid compress this tissue so as temporarily to diminish the amount of blood circulating in that portion, the sound produced is temporarily diminished or destroyed. He says that it has often occurred during his investigations that the appearance of the souffle was coincident with more or less extended movements of the fœtus, accompanied by a perceptible change in the conformation of the uterine tumor. He thus explains the different modifications to which he says this sound is subject. But without dwelling upon these shades of difference, let us see what value is to be attached to this sound according to the theory of M. Dubois, as a sign of pregnancy; or as an indication of the location of the placenta, and from which useful practical deductions may be drawn. In the first place, when it is the result of pregnancy, at what time does the vascular tissue of the uterus become so much developed, and when it is so favorably placed as to produce a sound sufficiently distinct to be heard by the observer? M. Dubois fixes the sixteenth or seventeenth week as the time when it may ordinarily be heard. But this must obviously depend upon a variety of circumstances, such af the depth of the pelvis, or the time when the uterus ascends into the abdominal cavity, the thickness of the abdominal parieties, &c., &c. There seem, notwithstanding the uncertainty which almost always attends the date of pregnancy, some well established cases in which the *bruit de souffle* was heard as early as the twelfth, and it is claimed by Depaul to have been heard at the tenth week; and it is highly probable that it may in most cases be heard before the conclusion of the fourth month. But in reference to its value as a sign of pregnancy, it is clear that if it exists in the vessels of the developed walls of the uterus, and not in the placenta, as has heretofore been generally supposed, as may occur from other causes than pregnancy, its value as a sign of this state is somewhat diminished. It has been heard, or a sound quite analogous, in cases where the uterus was distended with tumors, moles, and other morbid products. Hence, in diagnosing pregnancy, whilst it is one of the earliest, and certainly the most valuable of all the signs except the sounds of the fœtal heart, it must nevertheless be borne in mind that it is not of itself conclusive.

Again, it is readily perceived, form a knowledge of the anatomical structure of the uterus upon which it depends, that it can afford little or no evidence of the condition of the child occupying its cavity. Thus the fœtus may be feeble, or dead, and as the uterus, until its expulsion, would remain distended, and the same vascular activity continue, it would in nowise aid in establishing its viability or its maladies. When this sound was supposed to depend upon the utero-placental circulation, it was esteemed of some value in diagnosing twins, by establishing the existence of two placenta when the sound could be heard at two points remote from each other. With this transfer of the source of this sound from the placenta to the uterus, it is plain all hope of determining the condition of the former organ, its structural alterations and pathological changes, must cease. I cannot, however, but believe, from the best consideration which I have been able to give this subject, that, as the vascular system of the uterus is known to be most developed in the region of the attachment of the placenta, this point will be indicated by the *maximum* of sound corresponding thereto. This will be of great practical value in cases of placenta prævia, and in avoiding the more vascular part of the uterus, and the increased substance to be divided in the performance of the Cesarean section. Your friend,

JAMES P. WHITE.

